# Patient satisfaction with post-operative surgical services and associated factors at Addis Ababa City government tertiary hospitals’ surgical ward, cross-sectional study, 2022

**DOI:** 10.11604/pamj.2023.45.189.38416

**Published:** 2023-08-30

**Authors:** Ermias Algawork Kibru, Yidnekachew Girma Mogessie, Abel Alemayehu Regassa, Kirubel Tesfaye Hailu

**Affiliations:** 1 Addis Ababa University, College of Health Science, Addis Ababa, Ethiopia,; 2 Johns Hopkins Bloomberg School of Public Health, Baltimore MD, USA,; 3 Johns Hopkins Carey Business School Baltimore MD, USA, St Paul's Hospital Millennium Medical College, Addis Ababa, Ethiopia,; 4 Jimma University, Institute of Health, Faculty of Medical Science, Jimma, Ethiopia

**Keywords:** Patient satisfaction, surgical ward services, information provision, post-operative complications

## Abstract

**Introduction:**

patient satisfaction is an attitude resulting from a person's general orientation towards the total experience of health care. The study was done with the aim of evaluating post-operative patient satisfaction level with the quality of service provided at the surgical wards; determining how much factors related to physicians, nursing, laboratory, and information provision service influence satisfaction level; and determining factors impacting patient satisfaction level.

**Methods:**

a hospital-based quantitative cross-sectional study design was conducted in six Addis Ababa City Government tertiary hospitals from November 4^th^ to December 13^th^, 2022. Patients who had major operations done at the government hospitals from November 21^st^ to December 5^th^, 2022, were included in the study population. A pre-tested, structured, and Amharic-version questionnaire was used to interview patients. A bivariate and multivariate logistic regression model was used to identify the variables that had an association with the dependent variable. P-values less than 0.05 were considered statistically significant.

**Results:**

a total of 287 patients participated in the research, providing a response rate of 95%. Of the total participants, 144 were males (50.8%) and 143 were females (49.5%). The overall patient satisfaction level with surgical ward service is 96.2%. The level of patient satisfaction with nursing services is 94.8%, with physician's services it is 98.6%, with the facility it is 92.3%, and with the provision of information about post-operative complications it is 69.7%. Those who have above-first-degree educational status are less satisfied (66.7%) than patients with other levels of educational status. Only the patients' residency showed a small level of association (r= 0.145, p=0.014) with overall patient satisfaction with surgical ward service among the demographic components. The two variables that are strongly correlated with patient satisfaction are the adequacy of the time ward nurses spent with patients during evaluation and treatment (r = 0.503, p = 0.000) and adequate nurses' response to patients' calls (r = 0.498, p = 0.000). Post-operative patient satisfaction with surgical ward nursing service, physician service, hospital facilities, and the provision of information about post-op complications explain about 40.9 percent of the variation in the overall patient satisfaction with post-op care provided at the surgical ward. Patient satisfaction with nursing service has more significant effect with overall patient satisfaction than the other variables (β = 0.266, p =0.0002).

**Conclusion:**

post-operative patients at Addis Ababa City Government Tertiary Hospitals expressed a very high degree of satisfaction with the care they received in the surgical wards. The study also found that patients were generally less satisfied with the information they were given on drugs, side effects, and available treatment options. Another factor identified in the study that caused unhappiness was the unavailability of some pharmacy and laboratory services.

## Introduction

Patient satisfaction is multifaceted and a very challenging outcome to define. It seems easy to understand but hard to define. Satisfaction is not a pre-existing phenomenon waiting to be measured, but rather a judgment people make reflecting their experience under specific circumstances. A simple and practical definition of satisfaction would be the degree to which desired goals have been achieved [1]. Patient satisfaction is important as it will result in patient participation in health care and treatment affairs. Nowadays, patient satisfaction in health centers is recognized as a key indicator reflecting the efficiency of the organization [2].

Traditionally, consumer satisfaction has been afforded a high level of importance in commercial and market research. Similarly, there has been a growing interest in the measurement of patients' satisfaction in health care research. A number of studies show that hospitals with more satisfied patients generally provide higher-quality care as measured by standard quality metrics [3]. A meta-analysis of 15 studies done by Muluget H *et al*. about patient satisfaction with nursing care in Ethiopia estimated the pooled level of patient satisfaction with nursing care in Ethiopia at 55.15% (95% CI: 47.35, 62.95). Based on the subgroup analysis, the estimated level of satisfaction in Addis Ababa was 54.24% (95% CI: 46.84, 61.65) [4]. According to this study, patient satisfaction was influenced by the patient's history of admission, residence, availability, and skill of the assigned nurse, as well as the presence of other diseases [4].

According to a cross-sectional study conducted at Jimma University Specialized Hospital by Woldeyohanes TR *et al*., 61.9% of patients were overall satisfied with their in-patient care. The patients in medical wards were less satisfied than those in other departments. Patients with no formal education and patients from rural areas were more satisfied than their counterparts. Most of the patients were dissatisfied with the nursing, pharmacy, and laboratory services, the level of health education given, and the communication and information they received about their illness. while others complain about the crowdedness of rooms, privacy, confidentiality, and restricted visiting hours [5].

Abera RG *et al*. conducted a cross-sectional study at Tikur Anbessa Specialized Hospital and found that 59.7% of patients were satisfied with the clinical laboratory services provided. Patients were highly dissatisfied with the location of the laboratory, latrine accessibility and availability, latrine cleanliness and comfort, the waiting time for specimen collection, and the whole availability of requested tests [6]. This study aims to assess patient satisfaction level with post-operative surgical services at Addis Ababa City Government Tertiary Hospitals. It will assess the relationship between patient satisfaction level with nursing services, physicians, facilities and information provision about post-operative complications.

## Methods

**Study design and period:** a hospital-based cross-sectional study was conducted in six surgical wards of Addis Ababa City Government Tertiary Hospitals from November 4^th^, 2018 to December 13 ^th^, 2022.

**Study setting:** this study was conducted at six Addis Ababa City government tertiary hospitals, the surgical wards of which were purposefully chosen out of the tertiary-level government hospitals found in Addis Ababa for reasons of accessibility and less bureaucratic red tape. These are Yekatit 12 Hospital and Medical College (Yekatit 12), Zewditu Memorial Hospital (Zewditu), Menelik II Hospital, Tikur Anbessa Specialized Hospital (TASH), St. Paul Millennium Medical College (SPMMC), and St. Peter Specialized Hospital (St. Peter), which are located in the nation's capital, Addis Ababa. These hospitals are among Ethiopia's largest referral and tertiary-level hospitals.

**Participants:** the study population includes all adult patients who had a major operation in one of the six hospitals listed above between November 21^st^ and December 5^th^. Based on information obtained from each hospital, the average number of surgeries performed during the data collection period is 250 in TASH and St. Paul and 125 in the remaining hospitals.

**Inclusion and exclusion criteria**

**Inclusion criteria:** patients aged ≥18 years and who had major surgery, patients who have been operated at least 2 days prior, patients who are voluntary to participate in the research

**Exclusion criteria:** patients aged <18 years, patient whose post-op duration is less than 2 days, Patients with post-op duration of greater than 2 days but unconscious, Patients who are not voluntary to participate in the research

**Data source/measurement:** the data was collected using pretested, structured questionnaires, with samples selected using simple random sampling. Primary data is used for the purpose of data collection in this study. The primary data is gathered using questionnaires from a randomly selected sample of individual post-operative patients found in surgical wards. A pre-tested, structured, and Amharic-version questionnaire was used to interview patients 24 hours after their operation and after they were fully awake. The questionnaire was pre-tested and verified to be valid in other research done in Gondar. To ensure the quality of the data collected, training was provided for the data collectors, and the investigators directed and monitored the whole data collection process for consistency, completeness, and accuracy.

**Study size:** sample size (n) is determined based on the assumption of 55% prevalence, expected margin of error (d) 0.05 at 95% confidence interval (Z8/2). n becomes 380. After correction formula (Nadj= n/1 + n/N) is employed to adjust for the total population of <10,000, the final sample size has become 302. Then, the sample size is going to be proportionally allocated to each hospital surgical ward. Systemic random sampling method is going to be used to select study subjects from the patients.

**Study variables**

**Independent variables:** socio-demographic variables: age, sex, address, educational background, marital status, monthly income level, general health status (co-morbid diseases), frequency of hospital visit, frequency of operation Factors related to postoperative management by nurses: skill, sympathy for patients, maintaining privacy, respect during communication, prompt response to patient call, information about patient health status or progress, clear information about the investigations, spending adequate time with patient during treatment, adequate care at night Factors related to postoperative management by physicians: confidence in the skill of physicians, showing a caring attitude, maintaining privacy, communicating in an understandable way, availability when needed Factors related to postoperative management by hospital facility: Cleanliness of wards/beds, cleanliness of bathing room, cleanliness of toilet, adequacy of food and water, quality of food, accessibility of pharmacy and laboratory, cost for medications and investigation Factors related to information provision about postoperative complications and treatment options: Pain, Post-operative nausea and vomiting, sore throat, discomfort, depression, hunger, thirst, bleeding, re-operation, and death

**Dependent variable:** proportion of patients who would say they were satisfied

**Statistical analysis:** the data obtained from the questionnaire were collected, stored, and analyzed using Epi data version 2.9 and SPSS version 23.0 software. A P-value of <0.05 was considered statistically significant. Categorical data obtained were summarized using counts and percentages while continuous variables were summarized using mean and median. Bivariate analysis with chi-square and Multivariate regressional analysis was done.

**Bias:** one potential source of bias in this cross-sectional study could be selection bias. If the study only involved patients who were willing to participate and complete the survey, there could be a selection bias towards patients who had more positive experiences with surgical services. Patients who have had negative experiences may choose not to participate or complete the survey, leading to an overrepresentation of positive responses.

**Efforts to address the potential source of bias:** to address this potential source of bias, the researchers could use a random sampling technique to select participants for the study. By randomly selecting participants from a larger pool of patients who have undergone surgery at the hospital, the study sample will be more representative of the entire patient population. The researchers could also use incentives to encourage participation from a broader range of patients and increase response rates. Additionally, the survey could be designed to include questions that capture both positive and negative experiences, so that the researchers can gain a full picture of patient satisfaction with the surgical services.

**Ethical considerations:** ethical clearance for the study was requested and obtained from Addis Ababa University, College of Health Science, School of Public Health research ethical committee with Institutional Review Board (IRB) number of 022/057/SPH. The objective of the study was explained to every participant, and it was only after they gave their full informed consent that the data was collected. No person was obliged to participate without their consent. Moreover, participants have been assured that no damage would be inflicted on them because of their participation in this particular study by explaining to them the apparent purpose of the study which is for academic purposes. Any information obtained is and will be kept confidential.

**Funding sources:** this research did not receive any specific grant from funding agencies in the public, commercial, or not-for-profit sectors.

## Results

### Socio-demographic profile of the respondents

A total of 287 patients participated in the research, providing a response rate of 95%. The data was collected from 6 Addis Ababa City Governmental Tertiary Hospitals. Of the total participants, 144 were males (50.8%) and 143 were females (49.2%). The mean age of the respondents is 40.42 ± 16.34 years. About 27.9% of the respondents have no formal education, while the rest of the respondents have attended at least primary level education. 40.8% of the respondents reported being in poor or very poor health status in the last 4 weeks before admission. The mean period of waiting for surgery is 2.6 ± 6.3 months with a maximum waiting period of 6 years and a minimum of 0 month - emergency. About 70% of the patients reside in urban areas, while about 22% come from rural areas. Nearly half of the participants are half-time or full-time workers, while about 1/5^th^ of the participants are unemployed (Table 1). The descriptive statistics for the variables are presented in Table 2. The average score from the 5-point Likert scale with 5 as “very satisfied” and 1 as “very dissatisfied” for all the variables was computed to show the proportion of the respondents that were either satisfied or dissatisfied with the items of the variables. Where the mean for the variable is more than half of the 5-point Likert scale (i.e., 2.5), the respondents are satisfied, and where the mean for the variable is less than half of the 5-point Likert scale (i.e., 2.5), the respondents are dissatisfied.

**Table 1:** socio-demographic characteristics of the respondents

Variables	Category	N	N%
**Sex**	Male	144	50.20%
	Female	143	49.80%
**Educational Background**	No formal education	80	27.90%
	Primary(1-8)	82	28.60%
	Secondary(9-12)	57	19.90%
	Below college diploma	11	3.80%
	College diploma	30	10.50%
	First Degree	24	8.40%
	Above First Degree	3	1.00%
**Marital Status**	Single	68	23.70%
	Married	203	70.70%
	Divorced/Separated	6	2.10%
	Widowed	10	3.50%
**General Health Condition in Last 4 weeks**	Very Good	30	10.50%
	Good	62	21.60%
	Fair	78	27.20%
	Poor	87	30.30%
	Very Poor	30	10.50%
**Residence**	Urban	202	70.40%
	Semi Urban	29	10.10%
	Rural	56	19.50%
**Working Status**	Retired	18	6.30%
	Unemployed	59	20.60%
	Full time Student	19	6.60%
	Home Maker	50	17.40%
	Full time / Part time Worker	141	49.10%
**Data Collection Site**	St Paul	74	25.80%
	St Peter	35	12.20%
	Menelik II	33	11.50%
	Yekatit 23	37	12.90%
	Zewditu	36	12.50%
	TASH	72	25.10%

**Table 2:** patient satisfaction level with socio-demographic information

		overall patient satisfaction Level			
		Dissatisfied		Satisfied	
		N	N %	N	N %
**Data collection site**	St Paul	2	2.70%	72	97.30%
	St Peter	2	5.70%	33	94.30%
	Menelik II	1	3.00%	32	97.00%
	Yekatit 23	1	2.70%	36	97.30%
	Zewditu	0	0.00%	36	100.00%
	TASH	5	6.90%	67	93.10%
**Gender**	Male	5	3.50%	139	96.50%
	Female	6	4.20%	137	95.80%
**Marital status**	Single	4	5.90%	64	94.10%
	Married	7	3.40%	196	96.60%
	Divorced/Separated	0	0.00%	6	100.00%
	Widowed	0	0.00%	10	100.00%
**Residence**	Urban	7	3.50%	195	96.50%
	Semi Urban	1	3.40%	28	96.60%
	Rural	3	5.40%	53	94.60%
**General health condition in Last 4 weeks**	Very Good	0	0.00%	30	100.00%
	Good	2	3.20%	60	96.80%
	Fair	4	5.10%	74	94.90%
	Poor	3	3.40%	84	96.60%
	Very Poor	2	6.70%	28	93.30%
**Working status**	Retired	0	0.00%	18	100.00%
	Unemployed	1	1.70%	58	98.30%
	Full time Student	2	10.50%	17	89.50%
	Home Maker	1	2.00%	49	98.00%
	Full time / Part time Worker	7	5.00%	134	95.00%
**Educational background**	No formal education	3	3.80%	77	96.20%
	Primary(1-8)	5	6.10%	77	93.90%
	Secondary(9-12)	1	1.80%	56	98.20%
	Below college diploma	0	0.00%	11	100.00%
	College diploma	1	3.30%	29	96.70%
	First Degree	0	0.00%	24	100.00%
	Above First Degree	1	33.30%	2	66.70%

### Outcome data

The overall patient satisfaction level with surgical ward service is 96.2%. The level of patient satisfaction with nursing service is 94.8%; with physician service, it is 98.6%; with the facility, it is 92.3%; and with post-op complication information provision, it is 69.7%.

### Main Results

Overall patient satisfaction was reported to be the highest in Zewditu Memorial Hospital (100%) and the lowest in TASH, with a satisfaction level of 93.1%. Males are more satisfied with surgical ward service than females (96.5% vs 95.8%). 96.6% and 96.5% of patients who come from semi-urban and urban areas respectively are satisfied with the overall service provision at the hospitals. 94.6% of those from rural areas expressed overall satisfaction. Those who have retired have a high level of satisfaction with the surgical ward service (100%). Students are less satisfied than patients with other employment statuses with 89.5% expressing satisfaction. Those who have above-first-degree educational status are less satisfied (66.7%) than patients with other levels of educational status (Table 2). All components of the independent variables related to patient satisfaction with nursing service, physicians, facilities, and information provision are moderately correlated to overall patient satisfaction based on bivariate correlation analysis (Pearson correlation coefficient ‘r’ between 0.3 - 0.4, p between 0.0000 - 0.0002), except for two variables. The two variables that are strongly correlated with overall patient satisfaction are the adequacy of time ward nurses spent with patients during evaluation and treatment (r =0.503, p = 0.000) and adequate nurses' response to a patient's call (r = 0.498, p = 0.000). On bivariate correlation analysis of the demographic information and overall patient satisfaction, no significant association was found between most of the socio-demographic variables except for residence. Patients' residency demonstrated a small level of association (r = 0.145, p = 0.014) with overall patient satisfaction with surgical ward service. As shown in Table 3 of the regression analysis, patient satisfaction with surgical ward nursing service, physician service, facilities, and the provision of post-op complication information explain about 40.9 percent of the variation in the overall patient satisfaction with post-operative care provided at the surgical ward. Patient satisfaction with nursing services has more significant effect on overall patient satisfaction than the other variables (β = 0.266, p =0.0002).

**Table 3:** regression analysis tables

Model Summary				
**Model**	**R**	**R Square**	**Adjusted R Square**	**Std. Error of the Estimate**
1	.640a	.409	.401	.66701

Predictors: (constant), patient satisfaction with facility, patient satisfaction with physicians service, patient satisfaction with post-op complication information provision, patient satisfaction with nursing service dependent variable: overall patient satisfaction level

## Discussion

The study found that the overall patient satisfaction level with the surgical ward's post-operative care as 96.2%. The result is closer to satisfaction levels found in some studies [7-11] which were greater than 90% but higher when compared to most studies done in Ethiopia and worldwide [3,5,12,13]. This could be due to the fact that the study was conducted on recent post-op patients who have seen some improvement from their previous health condition, leading them to rate the service with a positive attitude, as shown in some studies [14].

The study found that the level of surgical ward patient satisfaction with nursing service was 94.8%, physician service was 98.6%, and satisfaction with the facility was 92.3%. The results indicated a high level of patient satisfaction that was also in line with the overall surgical ward postop patient satisfaction level. The results are higher when compared to previous studies done [6,11,15]. This could also be attributed to the hopeful attitude of the recovering postop patients. The lowest satisfaction level was found with regard to the provision of information about postop complications (69.7%). The level of satisfaction found was lower when compared to findings in other studies [3,11]. The study also found males were slightly more satisfied with the surgical ward service (96.5%) vs. females (95.8%). This result was in contrast with a study in Gondar, which found females more satisfied than males (93.6% vs. 88%) [8], and also with a study in TASH, which found females more satisfied with services than males [9]. Patients with educational status above the first degree were found to be less satisfied with the surgical ward service than others (66.7%), which is also in line with another study in Jimma that found satisfaction levels decreasing as educational status increased [5]. The study found patients from urban area are more satisfied than those from rural area. This was in contrast to the result found in the Jimma study, which stated that patients in rural areas were more satisfied [5].

The study found no significant association between socio-demographic information like age, gender, educational status, marital status, and working status with overall patient satisfaction with surgical ward post-operative care. Different studies support no significant association, whereas others found some association between some of the socio-demographic variables and patient satisfaction level [7,9,13,16]. Literature appears mixed on the importance of patients' demographic and social factors in determining satisfaction. Nevertheless, the literature does shed some light on how particular demographic factors affect patient satisfaction. This study found a significant association between residence and overall patient satisfaction. The study found no significant association between the frequency of surgeries and waiting period of surgery with post-op surgical ward patient satisfaction level. This result is in contrast to another study in eastern Ethiopia, which found a negative association between the length of the waiting period for outpatient services and satisfaction level [13]. This may be due to the participants of the study, being post-op patients, who give more significance to their surgery completion than the waiting period they spent.

The study shows patient satisfaction with physicians, nurses, facilities, and information provision contributes about 40.9% of the variance in overall patient satisfaction with surgical ward services. The components of those services show a significant association with overall patient satisfaction. In our study, the major areas of patient dissatisfaction in post-operative patient management are: by nurses, the provision of information about the importance of investigation (12.5%), the provision of information about side effects of medications (13.2%); by physicians, the availability of the responsible physician when needed; by facilities, the cleanliness of the latrines (17.8%), the adequacy and quality of food and water (15%), the accessibility of pharmacy and laboratory facilities (22%), and the fairness of medication and investigation costs (16.4%). These dissatisfaction levels are similar to other studies conducted in Gondar [14]. But the results are lower than a similar study in Jimma [5].

When compared to the other factors, this study found a high level of patient dissatisfaction with the provision of information about post-operative complications. The results show patient dissatisfaction with the provision of information about the risk of reoperation and death after operation (38%), the risk of depression and treatment options after operation (36.6%), the risk of hunger and thirst after operation (36.5%), the risk of discomfort and relieving methods after operation (35.2%), the risk of bleeding and treatment options after operation (33.8%), and the risk of sore throat and treatment options after operation (31.4%). The results were significantly higher than similar results in other studies [3,11].

**Limitations:** while the study on post-operative care provides valuable insights into the recovery process, the small sample size and narrow focus of the study limit its generalizability and do not provide a comprehensive understanding of the entire surgical care experience. Therefore, future studies should include larger sample sizes and explore the full range of experiences involved in surgical care, including pre-operative preparation, the surgical procedure itself, and the post-operative recovery period. This would provide a more holistic view of the factors that influence patients' overall satisfaction with their surgical care and help to identify areas for improvement in the delivery of healthcare services.

**Generalisability:** of the findings from this cross-sectional study on patient satisfaction with post-operative surgical services at tertiary hospitals in Addis Ababa may be limited to similar contexts. The study was conducted in government tertiary hospitals in a specific geographic location, and the results may not be applicable to different cultural or socio-economic settings. The sample size may also have implications for the study's generalizability, and results may not be representative of all patients undergoing surgical procedures in the country or region. However, the study's use of validated instruments and structured interviews enhances the credibility and transferability of the findings, and the results provide valuable insights into the factors influencing patient satisfaction with post-operative surgical services in similar settings.

## Conclusion

The study revealed a high level of patient satisfaction in Addis Ababa City Government Tertiary Hospitals' surgical ward. The study found a relatively lower level of patient satisfaction with the provision of information about medications, complications, and treatment options. The study also found some level of patient dissatisfaction with the availability of pharmacy and laboratory facilities in the hospitals.

### What is known about this topic

- *Ensuring high level of patient satisfaction is crucial for positive patient-hospital relationship;*
- *Several factors influence patient satisfaction level*.

### What this study adds

- *Revealed high level of patient satisfaction despite hospitals being resource limited;*
- *Identify areas of improvement that need attention*.
